# PRISM CI: A SOFTWARE SYSTEM TO SUPPORT AGING ADULTS WITH A COGNITIVE IMPAIRMENT

**DOI:** 10.1093/geroni/igad104.0317

**Published:** 2023-12-21

**Authors:** Sara Czaja, Francesca Falzarano, Darby Lucius-Milliman, Marco Ceruso

**Affiliations:** Weill Cornell Medicine, New York City, New York, United States; Weill Cornell Medicine, New York City, New York, United States; Weill Cornell medicine, New York City, New York, United States; Weill Cornell Medicine, New York City, New York, United States

## Abstract

Maintaining social connections and engaging in activities can become challenging for aging adults with a cognitive impairment (CI), which can result in deleterious cognitive, emotional, and physical health outcomes. This presentation will discuss the PRISM-CI pilot trial, which aims to examine the feasibility and potential efficacy of the PRISM-CI software system on enhancing connectivity and quality of life among a diverse sample of 50 older adults aged 65 and over with a CI. PRISM-CI, adapted from the PRISM system (developed by the Center for Research and Education on Aging and Technology Enhancement) for this population, is intended to support social engagement, memory, and access to resources and information. The sample includes 42 community dwelling individuals aged 65+ (M= 75.5, SD=6.78) with a cognitive impairment. Participants had access to the system for 5 months. We will present data regarding training challenges encountered by our participants, such as the need for more personalized training, and perceptions of usability and the perceived value of the various PRISM-CI features as well as suggestions for improvements (e.g., streaming services such as Spotify, e-Library services). The challenges of implementing PRISM-CI during the COVID-19 pandemic will also be discussed. In addition, preliminary data regarding the impact of the use of PRISM-CI on social isolation and loneliness will be presented. Overall, the findings suggest that use of PRISM-CI was beneficial and enhanced social support and connectivity. We will also discuss how user feedback can be leveraged to inform adaptions to enhance the efficacy and acceptability of technology applications.

